# The impact of children’s enrollment restrictions on household consumption: evidence from the migrant population

**DOI:** 10.3389/fpubh.2025.1529716

**Published:** 2025-04-28

**Authors:** Jianfeng Xie, Meiyi Guo, Chengyu Li

**Affiliations:** College of Business Administration, Liaoning Technical University, Huludao, China

**Keywords:** migrant, household consumption, migrant children, peer effects, state policy, public education, well-being, heterogeneity

## Abstract

**Introduction:**

This study explores the economic effects of school enrollment restrictions derived from the household registration system on the consumption behavior of migrant families. With intensified globalization and increasing population mobility, enrollment restrictions—arising from factors such as migrant status, policies, or resource allocation—have become a critical issue at the regional level. The effect of education policies on migrant children’s enrollment restrictions and household consumption willingness remains a subject of debate in the academic community.

**Methods:**

Using 2014–2017 household microdata of the migrant population, this study constructs an index of enrollment threshold for migrant children based on policy documents of each city. A microeconometric model is built to estimate and examine the economic effects of education policies for migrant children on household consumption levels.

**Results:**

The results indicate that a decline in the school enrollment threshold can significantly improve the household consumption of migrant families. The findings pass several robustness tests and endogeneity tests. Furthermore, the impact of the schooling threshold on household consumption is more profound among migrant households with characteristics such as being an only child, intra-provincial mobility, and a willingness to settle down.

**Discussion:**

The conclusion highlights that regional educational equity can effectively promote the consumption willingness of the migrant population and enhance socioeconomic inclusivity. The study provides important policy recommendations for safeguarding and supporting the well-being of migrant families.

## 1 Introduction

With the expansion of the population migration scale, by the end of 2020, China had 376 million migrant population, accounting for 26.03% of the total population, an increase of 69.73% compared to 2010 (1). China has steadily entered the era of population mobility, with the migrant population becoming an important force in the China’s labor market (2). However, as China’s economic development and urbanization are entering a new phase, the consumption characteristics of the migrant population are quietly changing. Due to external factors such as China’s dual urban–rural dual structure and household registration system, there is an imbalance between the public services and social security enjoyed by the migrant population and urban residents, leading to different consumption levels and characteristics between the two (3). Compared to urban residents, the migrant population has a higher household savings rate and a lower consumption tendency (4). This is especially true when the willingness of the migrant population to return is strong, which may be constrained by factors such as the household registration system or educational barriers (5, 6), leaving a considerable portion of the consumption potential of the migrant population unleashed. China’s new urbanization, as an important foundation for smooth domestic circulation, emphasizes a people-centered approach. It aims to ultimately achieve the urbanization of the agricultural transfer population, allowing the floating population to enjoy the same social treatment as local citizens in the host country. Therefore, exploring the influencing factors of the consumption level of the migrant population not only enable us better understand the well-being of China’s migrant population but also contributes to addressing and improving the social welfare issues of the migrant population from a fundamental perspective.

Improving the growth environment for migrant children is also an important way to enhance the well-being of the migrant population. By the end of 2022, the number of children of China’s migrant population is approximately 130 million, accounting for 46.4% of the total child population in China, and nearly half of country’s children are affected by population mobility (7). Current studies have shown that school-age children who migrate with their parents benefit from parental accompaniment, allowing them to experience a superior home learning environment (8–12). Additionally, a richer educational environment will serve as a positive factor in children’s development (13–15). China’s Compulsory Education Law mandates that all children must receive nine 9 years of education. However, due to educational policies and institutional barriers in host cities, children of migrant populations may face difficulties in enrolling in local public schools. As a result, they often have no choice but to attend schools specifically established for the children of migrant workers in urban areas (16, 17), or even leave them in their hometowns for education. Such admission criteria set by local governments for non-registered migrant children to enroll in public schools are defined as “school entry thresholds.” These thresholds impose various enrollment conditions, requiring specific documents and proof of eligibility, thereby limiting the number of migrant children admitted to public schools (18). It has been noted that there is a certain educational model gap between the children of migrant populations and local children in the cities they move to (9). As a result, the educational expectations of migrant families often influence the allocation of economic resources within the household (19). And for lower-income families or vulnerable groups, the cost of raising children for the purpose of increasing the children’s educational experience will outweigh the family’s savings motivation (20, 21), further depleting their limited resources (22). However, when structured opportunities are available, low-income families tend to exhibit higher savings levels (23).

The Central People’s Government has consistently placed a high priority on the compulsory education of migrant children. To this day, the division of fiscal expenditure responsibilities and transfer payments in China are based on the premise of a non-mobile population, allocating educational resources based on the registered population in each jurisdiction. However, the spatial distribution of the population is becoming increasingly concentrated, and the trend of population migration between regions remains significant, so that basic public education has not yet achieved an equal distribution based on the resident population (24, 25). The differences in social security services caused by the urban–rural resource allocation have led to distinct perceptions of social status changes among groups such as migrant populations and registered immigrants (26). This situation makes them more aware of the instability of the job market and the vulnerable position of family members who move with them, which makes family mobility limited (27). When migrant populations face unstable expected income, increased career contributions, and poor mobility, their longing for a stable future leads them to increase savings and remit money back to their hometowns to mitigate the income risks for the entire family (28), which inevitably reduces the level of their consumption in the destination locations (29). It is evident that the supply of local educational resources is an important factor influencing the consumption behavior of migrant families. However, what specific impacts does it have? Are there differences among migrant families with varying characteristics? Exploring these questions holds significant policy and economic implications for promoting equity in public education for migrant children and enhancing the well-being of migrant populations. In 2024, the latest meeting report from the Central Committee of the Communist Party of China emphasized improvement of the institutional mechanism for promoting a new type of urbanization, and the basic public services that promote the enjoyment of the same rights as the household population include compulsory education for migrant children (30). Therefore, this article examines the economic effects on migrant families from the perspective of education for migrant children, which is of great significance for improving the quality of life of the migrant population and enhancing people’s well-being, and for promoting the new urbanization strategy centered on people.

A review of the literature and policy measures suggests that public education investment may have either a promoting or inhibiting effect on household consumption. However, existing studies primarily focus on macro-level data and the general population, with limited research examining migrant households’ consumption behavior using micro-level data. In particular, no studies have explored whether compulsory education investment, as reflected in school entry thresholds, leads to a crowding-in or crowding-out effect on the consumption of migrant households at the micro level. Therefore, this paper investigates the economic impact of migrant children’s education on migrant households. This research is of great significance for improving the quality of life of migrant populations, enhancing public well-being, and advancing the human-centered strategy of new urbanization. For a long time, receiving education in public schools at the destination has been a major challenge faced by the children of migrant populations, which will inevitably have a certain impact on the economic decision-making of the family. Based on this, this study focuses on examining the impact of enrollment thresholds for Chinese migrant children during the compulsory education stage on the consumption levels of migrant families. In terms of empirical data, this paper references studies by Wang Ru et al. (31) and Zou et al. (32) to construct and calculate the enrollment thresholds for compulsory education. These thresholds are matched with the national dynamic monitoring survey data of the migrant population (CMDS), and constructs a micro-database reflecting the characteristics of migrant families and their consumption situation in cities with different enrollment thresholds, which provides a data foundation for the subsequent research.

The research results of this paper find that the reduction of education access thresholds during the compulsory education stage has a significant promoting effect on the overall consumption levels of migrant families. This effect shows differential impacts in multiple dimensions. Compared to other groups, families that migrate within the province, families with only one child, families with a willingness to settle, families whose children do not migrate with them, households with low-and-middle income levels, and families where the head of household is employed in the non-public sector experience more pronounced and far-reaching effects on their consumption. The findings reveal the potential mechanism of education policy adjustment on the economic behavior of households, and provide important empirical evidence for further optimizing the allocation of education resources, promoting consumption growth and socio-economic inclusive development.

The potential marginal contributions of this study lie in the following three aspects. First, it expands the research perspective beyond previous studies, which have primarily focused on the impact of higher education policies on migrant households. This paper shifts the focus to the critical stage of basic education and examines the well-being of migrant households through the policy window of migrant children’s access to primary and secondary education. By systematically exploring the micro-level effects of local government education policies on improving the well-being of migrant populations, this study provides a new causal identification framework for understanding the factors influencing migrant household consumption. Second, it innovatively constructs and quantifies the School Entry Threshold Index for migrant children using the Analytic Hierarchy Process (AHP), breaking away from the traditional binary policy evaluation framework. By employing AHP to measure the difficulty of school enrollment across different regions, this study provides a more precise assessment of the impact of school entry barriers on migrant household consumption. Third, it further supplements the understanding of the social heterogeneity of policy effects. Against the backdrop of global efforts to enhance migrant social integration and protect their legal rights, this study delves into the diverse characteristics of the migrant population. It explores how public education policies implicitly generate differentiated impacts on social identities and demographic groups, analyzing variations in risk perception sensitivity within the migrant population. These insights offer valuable reference points for the formulation of inclusive and targeted education policies.

The remaining structure of this paper includes: Section 2 presents the key facts; Section 3 provides a literature review; Section 4 describes the data and model design; Section 5 reports the baseline empirical results; Section 6 conducts robustness checks and endogeneity tests; Section 7 explores heterogeneity and mechanism analysis; and finally, conclusions and policy implications.

## 2 Typical facts about the education of migrant children in China

### 2.1 Policies on the education of migrant children

**Figure 1:**
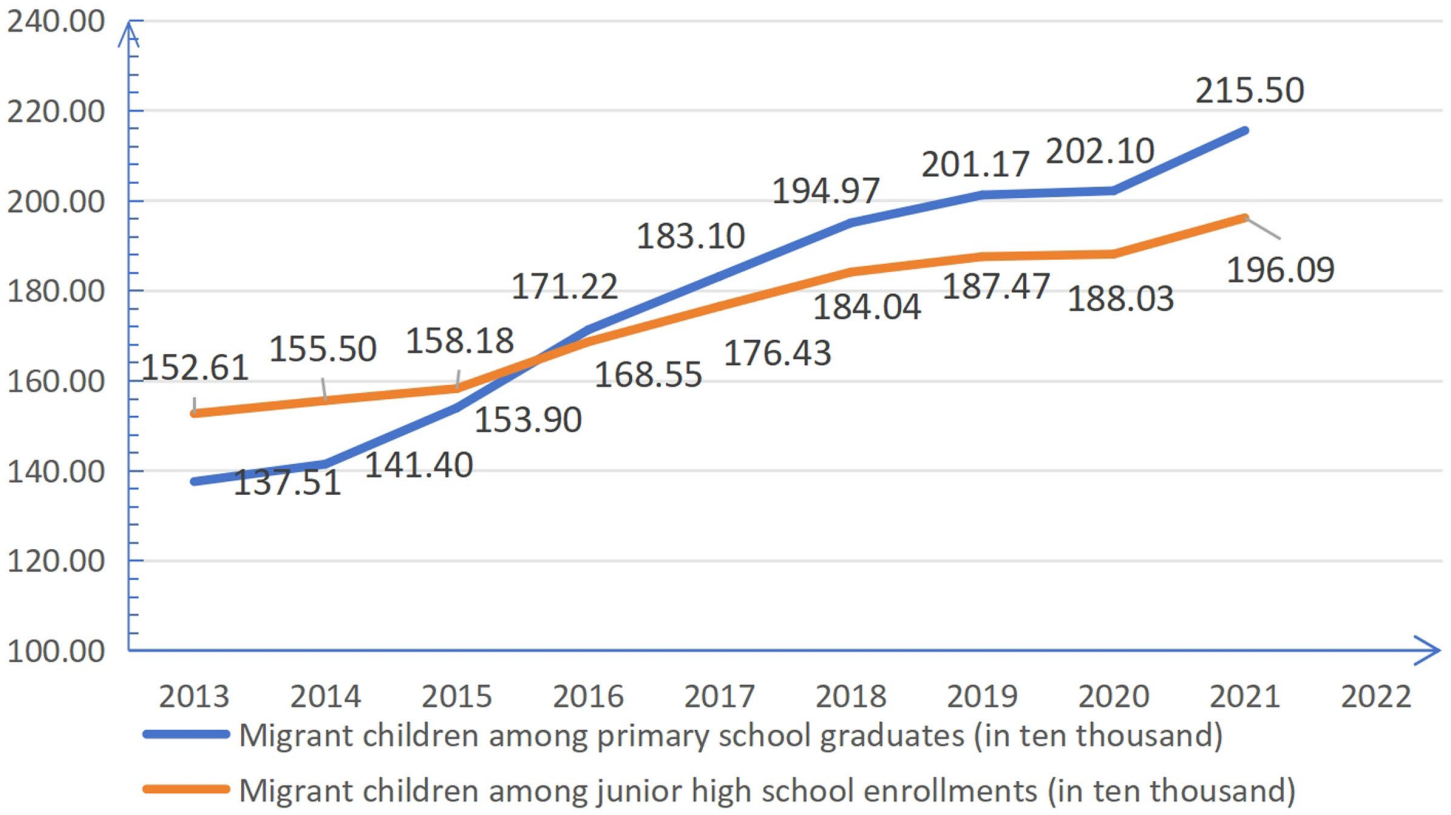
The number and comparison of migrant children among primary school graduates and junior high school enrollments from 2013 to 2020.

The education policy for migrant children has evolved through several stages: from “two priorities” in 2001, to “two incorporations “in 2014, and then to “two unifications” in 2016. This progression aims to maintain fairness in the supply of compulsory education and the orderly allocation of educational resources (24, 33). As shown in Figure 1, from 2016 to 2021, the number of migrant children graduating from primary school exceeded those enrolling in junior high school, with the gap gradually widening. In 2021, nearly 194,100 migrant children chose to return to their hometowns for enrollment at the transition from primary to junior high school (34). This reflects that enrollment for migrant children still faces challenges. From the perspective of the conflict between educational supply and demand, these issues are not only due to insufficient total supply but also largely stem from the mismatch in the spatial distribution of public education resources. This mismatch makes it difficult for existing educational resources to meet the changing demands of compulsory education caused by population migration, resulting in inefficient overall allocation of educational resources.

To this day, the central government’s policy approach regarding the education of migrant children still emphasizes addressing the enrollment issues of all migrant children as much as possible. However, local governments also face the potential problem of overcrowded educational resources. Therefore, under the dual pressure from both sides, local governments ease the enrollment congestion caused by supply and demand imbalances by setting enrollment criteria (35). The enrollment policy for migrant children, as a differentiated policy implemented by governments of various receiving areas for migrant children and local registered school-age children, includes specific measures such as policies for enrollment during the compulsory education stage, policies for the high school entrance exam in other regions, and policies for the college entrance exam in other regions (32).

### 2.2 Implementation policies for the education of migrant children

From the perspective of regional economics, the implementation of migrant children’s education policies varies significantly across different cities. This reflects the diverse urbanization processes, migrant population dynamics, and public resource allocation strategies in different regions. Taking Wenzhou, Shenzhen, and Shanghai as examples, each city has distinct characteristics in policy design, implementation outcomes, and regional economic logic. Wenzhou, driven by its strong private economy, has attracted many migrant worker families to settle. The city has pioneered flexible policies such as “zero-threshold enrollment” and a “point-based admission system,” breaking the restrictions of residence permits and significantly increasing the proportion of migrant children in public schools, reaching over 90% by 2024.Shenzhen’s education policy aims to support industrial upgrading through educational equalization. With a high proportion of public school seats and a “three-in-one” management model, the city ensures equal enrollment rights for migrant children while dynamically adjusting to population inflows and the resulting educational pressure. In contrast, Shanghai has adopted stricter restrictions on migrant children’s education policies. As a mega-city, it follows a resource-intensive strategy, setting high enrollment thresholds to balance public service provision with population pressure.

In China’s compulsory education stage, the enrollment policies for migrant children are influenced by the household registration (hukou) system. Different cities impose varying admission requirements for migrant children. According to policy regulations, migrant children must provide a set of documents to verify their guardianship, residence status, and parents’ employment conditions. Based on existing policy guidelines, the admission requirements for migrant children generally include four categories of documentation, as detailed in Table 1: 1. Proof of Relationship—Documents such as ID cards, household registration booklets, and family planning certificates to confirm the legal relationship between the child and their guardian. 2. Proof of Residence—Documents such as temporary residence permits, property ownership certificates, and residency verification from community offices to confirm the family’s actual place of residence. 3. Proof of Employment—Documents such as legal employment or business operation certificates and social security payment records to verify the parents’ employment status in the new location. 4. Other Supporting Documents—Items such as cross-regional school transfer permits issued by the original place of enrollment and student health records to ensure compliance with local school admission policies. This policy framework aims to regulate the eligibility of migrant children for school enrollment. However, due to variations in policy enforcement across different regions, it also reflects the differentiated approaches of local governments in managing urban public resources.

**Table 1:** Enrollment threshold indicator system for migrant children.

Goal layer	Criteria layer	Indicator layer
Enrollment threshold for migrant children	Relationship Proof A1	ID card, HuKou B1
		Proof of no guardianship at the household registration location B2
		Certificate of Birth Control, Marriage and Childbirth Certificate of Migrating Population B3
	Residence Proof A2	Temporal Residential Permit B4
		Residence Permit B5
		Proof of residence (community) B6
		Proof of rental or purchase B7
	Labor Proof A3	Proof of legal profession or business B8
		Proof of social security payment B9
	Other ProofA4	Student Status Certificate B10
		Letter of permission for study abroad from the original education department B11
		Birth Certificate、Vaccination Certificate B12
		Comprehensive Evaluation Handbook B13
		Other conditions B14

## 3 Literature review and policy background

### 3.1 Research on the consumption behavior of the migrant population

#### 3.1.1 Consumption behavior of the migrant population

In existing research, the migrant population exhibits differentiated consumption characteristics. Islam (28) found that immigrants in Australia have a higher savings tendency than local residents, while Gatina (36) argued that Australian immigrants save less compared to local residents. Other studies have shown that Hong Kong immigrants in Canada tend to have consumption habits similar to local residents (37). Vietnamese migrant workers’ families experience a significant consumption gap due to remittance behaviors (38). The consumption behavior of China’s migrant population shows a conservative characteristic of “low consumption, high savings,” with average consumption capacity lower than that of ordinary citizens (6). Consumption inequality mainly exists in categories such as household equipment, cultural and entertainment expenses, healthcare, and housing expenditures (39). Additionally, some studies have found that rural migrant families with urban residency tend to place more emphasis on conspicuous consumption compared to local rural families (40, 41). The new generation of Chinese immigrants are also more inclined to consume in urban areas (42).

Research on the factors influencing the consumption behavior of the migrant population can be mainly divided into three categories: urban economy, individual characteristics, and social characteristics. Affected by the urban–rural dual structure, China’s urban labor market exhibits a segmented characteristic, resulting in certain employment achievements and wage disparities between migrants and local populations (43). Although the efficiency of factor allocation in the urban labor market allows more family members to migrate to cities together (44), the accompanying rise in urban living costs, such as food and clothing, places pressure on family finances and migration decisions (45). Additionally, high housing prices and rents in the destination cities not only increase the living costs and working hours for migrants, raising their savings rates, but also increase the probability of children staying at home, weakening parental companionship and educational investment, which adversely affects children’s educational development (46, 47). More profoundly, this may reduce the degree of social integration and increase the push factors against incoming migrants (48, 49).

Another research direction focuses on analyzing the impact mechanism of the characteristics of the migrant population on consumption. Under the perspective of consumption theory, it has become a consensus that the consumption capacity of migrant population households is closely related to their income levels, and increasing the wages of migrant workers is seen as an effective way to release their consumption potential (50, 51). The improvement of the education level of the migrant population not only effectively raises the wage levels of migrant workers, allowing more educated technical migrants to have higher consumption levels (52), but also leads to consumption inequality among different groups (53). Additionally, it promotes the completeness of social insurance, especially for women with higher education levels, who demonstrate stronger competitiveness and preferences when employed in non-public sectors (54). At the same time, the migrant population is more susceptible to the influence of family age structure compared to local residents, particularly in the fields of education and healthcare consumption (55). For instance, the cost of raising children aged 7–15 in migrant worker families tends to be higher, primarily due to the educational system barriers faced in the cities they migrate to Luo and Li (29).

Migration patterns also have a profound impact on consumption decisions. Short-term migration can increase the motivation to save due to income uncertainty, thereby reducing consumption demand (56). Conversely, a stronger willingness to remain in the city amplifies the positive effect of wage growth on consumption levels (51). Long-term migration and residence significantly reduce the amount of remittances sent back home (57), which is beneficial for immigrants to invest and consume in their new location (58). It is noteworthy that the completeness of family migration and family size can also affects the migrants’ propensity to save and promotes their consumption in that city (28).

In addition to economic factors and individual characteristics, there are also studies that explore the impact of urban security factors on the consumption of the migrant population from a social perspective. Unlike the immigration situations in other countries, a key characteristic to focus on when discussing Chinese immigrants is the household registration system (hukou), which is closely related to the rights and social welfare of Chinese residents (59). When the household registration system imposes restrictions on social security and credit eligibility for the migrant population, it can lead to lower consumption levels in the host cities, with their marginal propensity to consume significantly lower than that of urban residents, in which the consumption structure is primarily limited to survival-oriented consumption (60, 61), and this consumption gap tends to widen over time (62).

At the same time, the improvement of the overall level of urban public services, such as social security, basic education, and pension guarantees, has been shown to have a significant income-increasing effect. It can have a long-term positive impact on household consumption levels and residential welfare through the willingness to stay and consumption peer effects (6, 63–65). By significantly increasing the coverage of social insurance, it can effectively alleviate the dual pressure of precautionary savings and liquidity constraints, creating favorable conditions for improving the consumption levels of low-income families (6, 66). Medical insurance, as one of the important components of social security, can significantly reduce household savings and promote consumption among the rural population through the New Cooperative Medical Scheme (NCMS) (67). Commercial health insurance can also promote consumption, especially for low-income, risk-neutral and other mobile populations, further facilitating the upgrading of consumption structures and addressing consumption inequality (68). Additionally, some studies have found that the moderating effect of housing security on consumption differences may weaken the positive impact of the migrant population on subjective well-being, revealing the moderating mechanism between housing security, consumption, and well-being (69).

In addition to government guarantees, the social environment of the inflow area also has a profound impact on the consumption behavior of the migrant population. The consumption behavior of urban residents has created a significant siphoning effect by compressing the social networks of the migrant population and stimulating their status-chasing motivation, exacerbating consumption inequality between the migrant population and local residents (70). Social factors such as peer environments and identity recognition also exert external effects, promoting residents’ consumption desires (71–73). This effect is particularly pronounced in education expenditure, which may lead to an excessively high proportion of education expenditure and even distort the balance of consumption structure (74). Finally, the dual social identity of the new generation of migrant workers (as both citizens and migrant workers) internalizes their consumption behavior. Social factors such as urban identity stimulate the consumption willingness of the new generation of migrant workers, further enriching the theoretical perspective on the consumption behavior of the migrant population (75).

#### 3.1.2 Consumption and well-being of the population

Marx once mentioned, “In addition to food, clothing and housing are the two major needs of humanity. People’s labor and productive life are all aimed at maintaining the needs of material survival (76).” This means that human survival and development cannot be separated from the needs of material life, which often restricts people’s spiritual production activities, and consumption primarily satisfies material needs. Basic goods can meet people’s fundamental needs, and the satisfaction of these basic needs greatly enhances individual happiness. Existing research has found a close relationship between happiness and material consumption. In discussions about happiness economics, it has been found that Adam Smith (77) consciously linked the living standards of workers to happiness (78). Noll and Weick (79) empirically discovered a positive correlation between consumption and subjective well-being, indicating that consumption can provide a solid material foundation for people to pursue a happy life. According to neoclassical consumption theory, rational behavior means maximizing one’s own utility or welfare, and the effective form of satisfying the utility of rational economic agents comes from increased consumption (80). Therefore, in this study, exploring the happiness of migrant families in relation to household consumption is of great significance for enabling migrants to settle and thrive in their new locations and to unleash their consumption potential.

As one of the “three engines” driving economic growth in macroeconomics, consumption in China is currently facing a situation of long-term insufficient demand development. This could lead to a series of negative impacts, such as overcapacity, over-reliance on investment and export-driven, ultimately hindering the sustained growth of our economy. From the perspective of China’s urban–rural dual structure, in addition to the general interpretation in Western economics, the formation of this structure is also attributed to the uniqueness of China’s history and system. It is not only confined to the market economy level but also reflects in many aspects such as society and culture. From the reform and opening up to the present, China has made significant achievements in the process of improving the socialist market economic system, but achieving great achievements in a short period has also brought certain challenges and problems. Among them, a “new dual structure” has been formed between the registered population and the non-registered population in the same city (81). One manifestation of this is the uneven distribution of public resources caused by the household registration system. Migrant populations face economic, social, cultural challenges and identification dilemmas, finding it difficult to enjoy the same treatment as registered residents, which is exemplified in the obstacles to school enrollment for their children moving into the city.

To explore the impact of China’s structural factors on residents’ consumption behavior, this paper focuses on the consumption issues of migrant populations caused by the differences in public education resources due to the household registration system. The policy implication is that if the educational resources for registered and migrant populations are balanced, it will help stimulate consumption and improve residents’ quality of life. This study concentrates on family consumption and welfare in the context of educational opportunities for migrant children. The results show that increasing educational opportunities for migrant children brings dual benefits to both migrant families and urban development. On the one hand, it can release the consumption potential of families, helping to increase family consumption levels and stimulate urban economic vitality. On the other hand, it contributes to the accumulation of human capital for children (82), playing a positive role in both the future quality of life for families and the long-term human capital structure of cities.

### 3.2 Research on the economic effects of education policies for migrant children

Evaluating whether public education allocation can lead to wiser economic decisions for households is an important perspective for exploring the quality of life of the migrant population. Firstly, against the backdrop of insufficient political incentives for local governments, public education investment across China is influenced by transfer payments and the substitution effect of government financial resources, failing to effectively align with the goal of educational equity (83). This necessitates that the central government adopt proactive strategies or decentralize power to encourage local governments to actively implement these goals (84, 85). As a result, children of the migrant population still face enrollment threshold issues during the compulsory education stage, with high thresholds leading to a halving of migration rates, particularly among low-income groups (86).

Investing in children’s education can reverse the negative impact of genetics, family environment, and social environment on their education (87). A good family economic situation often provides children with richer educational resources, meeting the needs for resources to develop their cognitive and personal skills. Influenced by the traditional expectation of Chinese parents for their children to succeed, parents are more inclined to choose stable jobs or participate in social security in the destination area early on to meet enrollment requirements for their children, which may lead to labor skill discrimination, exacerbating the unequal distribution of educational resources and fairness losses within the migrant population (88). On the other hand, education policies have a guiding effect on the migration decisions of the migrant population, meaning that regions with more lenient education policies positively influence migration decisions and city choices (89–91). This enhances the attractiveness of policy reform areas, forming a “depression effect” (35), and even affects the risk of left-behind children from rural households (31).

Additionally, some scholars have found that government education policies influence household consumption expenditures. According to the human capital theory proposed by Gary Becker, the expected income effect suggests that the expansion of education policies reduces household education costs. On one hand, families anticipate higher future earnings for their children, boosting consumer confidence and leading to a “crowding-out effect” (53). On the other hand, welfare policies such as compulsory education (free public education) increase household expectations for children’s education, prompting greater investment in education and resulting in a “crowding-in effect.” Based on the Life-Cycle Hypothesis, improvements in both the quantity and quality of education brought about by education policies can significantly enhance children’s future earning capacity. This, in turn, reduces households’ precautionary savings and increases their current consumption levels (92). Furthermore, according to the precautionary savings theory (93), families often tend to increase savings and reduce current consumption due to the prolonged duration of children’s education and the associated future financial burdens. In this context, public education guarantees can effectively alleviate families’ concerns about future expenses, thereby reducing the need for precautionary savings. Moreover, Chen (94) argues that government fiscal expenditures on higher education significantly stimulate domestic demand.

Under different educational policy contexts, household consumption decisions often vary. Compulsory education, as a fundamental and long-term form of education for children, provides stability in educational policies. According to the Permanent Income Hypothesis, the stability of compulsory education policies enhances households’ long-term income expectations, leading to reduced savings and increased consumption (95). Higher education, being closely linked to children’s future earnings, influences household consumption through human capital investment decisions. According to the human capital investment model, educational expenses can be viewed as a discounted investment in children’s future income. Thus, the return on higher education plays a crucial role in determining consumption tendencies. Chen (96) found that although migrant families generally exhibit a strong willingness to invest in education, when college entrance exam (gaokao) policies for non-local students become more restrictive, households tend to reduce educational expenditures. This effect is particularly pronounced among economically disadvantaged families and those with multiple children.

Some studies have examined household consumption behavior from the perspective of peer effects, utilizing various theoretical frameworks. According to social comparison theory, individuals often evaluate their consumption choices by comparing themselves with others in their social group. As a result, parents are motivated by peer group behavior when making educational decisions for their children, often choosing similar or even higher-quality educational products. Some research approaches peer effects from the perspective of social preferences in behavioral economics. Studies have found that individuals consider not only their own needs and utility but also experience social comparison pressure or positive peer influence (97), leading to herd behavior (98). This occurs for two main reasons: Information Asymmetry in Education—Due to the widespread education competition (“education arms race”), parents often imitate the educational choices of surrounding families without fully understanding the actual benefits (99). Education as a Social Status Signal—Educational expenditures can serve as a signal of social status. Driven by social comparison and identity recognition needs, families tend to make similar decisions regarding their children’s education (100).

In terms of measuring the enrollment thresholds for migrating children, existing studies have mainly employed textual analysis, entropy method, and nonlinear principal component analysis (31, 32, 86, 90, 101) to confirm the economic effects of enrollment policies for migrating children. In existing research, there is no consensus on the impact of public education expenditure on consumption, with findings generally divided into “crowding out” and “crowding in” effects. Research groups focus on exploring the effects of public education expenditure on urban and rural residents. Compared with existing studies, this paper focuses on migrant families facing enrollment issues for their children, employing more rigorous quantitative methods and empirical analysis to investigate the consumption effects of families constrained by enrollment restrictions. Based on the above analysis, it proposes the hypothesis that lowering enrollment thresholds can enhance the consumption capacity of migrant families. Additionally, it considers and distinguishes the characteristics of the migrant population, examining the different consumption effects within this group in the subsequent regression analysis, providing more precise decision-making references for optimizing basic education supply and improving the welfare of the migrant population.

## 4 Data description and model design

### 4.1 Samples and data

This article uses data from the China Migrants Dynamic Survey (CMDS) conducted by the National Health Commission from 2014 to 2017 for verification. The survey covers areas with a high concentration of migrant populations across the country, with an annual sample size of nearly 200,000 households. It is currently the best representation of the basic situation, migration characteristics, and microdata on the impact of migrant populations on social and economic development in China. The CMDS defines the migrant population as individuals aged 15 to 59 who have resided in the destination area for more than 1 month and do not have a local household registration (hukou). This study selected 49 cities as research samples, based on the criteria of provincial capitals, economically developed cities, and cities with a population of over 1.5 million according to the 2017 China Urban Construction Statistical Yearbook. Specific details can be found in Table 2. Due to the impact of the compulsory education enrollment policy in China, only children within the compulsory education age and those who will receive compulsory education in the future are affected. Children who have already entered junior high school or high school will face subsequent enrollment policies (such as the high school entrance exam policy and the college entrance exam policy in other regions). Therefore, this study only retains samples of families with children who are below the age of 12, i.e., those who have not yet entered junior high school. Children in this age group are influenced by the enrollment criteria for compulsory education. To improve the consistency of the study and reduce significant differences in parental investment in children’s education in special family structures, such as divorced or widowed families, in order to make the research subjects more comparable, this study excluded samples of migrant populations who were divorced or widowed. The final dataset consists of 200,947 family samples from 2014 to 2017.The city-level data comes from the National Bureau of Statistics of China, the “China Statistical Yearbook,” the “China Urban Statistical Yearbook,” various provincial and municipal statistical yearbooks, and the CEIC database. The descriptive statistics of each variable are shown in Table 3.

**Table 2:** The list of 49 sample cities.

City types		Specific cities
Cities with an urban population exceeding 1.5 million	Provincial capitals	Shijiazhuang, Taiyuan, Hohhot, Shenyang, Changchun, Harbin, Nanjing, Hangzhou, Hefei, Fuzhou, Chengdu, Nanchang, Jinan, Zhengzhou, Wuhan, Changsha, Guangzhou, Nanning, Haikou, Guiyang, Kunming, Xi’an, Lanzhou, Xining, Yinchuan, Urumqi.
	Municipalities directly under the central government	Beijing, Tianjin, Shanghai, Chongqing.
	Other cities	Tangshan, Handan, Wuxi, Xuzhou, Changzhou, Qingdao, Dongguan, Shenzhen, Foshan, Suzhou, Ningbo, Jinhua, Wenzhou, Dalian, Xiamen, Zibo, Yantai, Linyi, Luoyang.

The cities are categorized based on provincial capitals, municipalities directly under the central government, and cities with a population of over 1.5 million according to the 2017 China Urban Construction Statistical Yearbook.

**Table 3:** Results of descriptive statistics.

Variable	Mean	Std. Dev.	Min	Max
Total household consumption (Yuan/month)	4083.136	3197.148	0	367,000
Age of children	5.591088	3.557789	0	12
School enrollment threshold	0.8867577	0.5461831	0.1273	2.6538
Gender of household head	0.5192338	0.4996312	0	1
Age of household head	33.96092	6.497747	22	74
Ethnicity of household head	1.060807	0.2389761	1	2
Age of spouse	33.97152	6.471545	20	87
Household registration status of spouse	1.238634	0.510056	1	3
Household size	3.236847	0.9418424	1	10
Government education expenditure	0.122918	0.0694911	0.0013353	0.4282025
Gross Domestic Product Growth Rate	8.857005	1.97991	0	16
Real GDP per capita (Yuan)	90881.76	28787.69	3337.1	167,411
Second Industry Ratio (%)	38.36334	11.25948	0	62.1

### 4.2 Variable selection

#### 4.2.1 Dependent variable

The core explained variable of this article is total household consumption. In this article, each household is selected as a sample, and total household consumption expenditure is selected, with the variable logarithmically treated in the regression analysis. Additionally, since the regression results may be influenced by extreme values from micro-level subjects, housing consumption, and all-inclusive living expenses, robustness checks are conducted using variables ex-housing consumption, per capita consumption, and all-inclusive living expenses as explained variables. The core dependent variable of this study is household total consumption. Each household is treated as a single observation, and total household consumption expenditure is selected as the key variable. In the baseline regression analysis, the total consumption expenditure is transformed using the natural logarithm. To ensure the robustness of the regression results, adjustments are made to account for potential biases caused by extreme values at the micro level, housing expenditures, and in-kind benefits such as meals and accommodation. Specifically, three alternative dependent variables are used for robustness checks: (1) an adjusted consumption measure excluding housing expenditures, (2) per capita consumption, and (3) a consumption measure that includes in-kind benefits. These robustness tests help verify whether the core conclusions are sensitive to the specificity of housing consumption, household size effects, and the conversion methods for in-kind consumption, ensuring the reliability of the study’s findings.

#### 4.2.2 Independent variable

The core explanatory variables are the enrollment threshold (entrance_index), child age (child_age), and their interaction term. The standardized definition of the threshold for enrollment of migrant children requires a uniform and intertemporal threshold index for comparison. The construction of the evaluation system serves as the starting point and premise for measuring the enrollment threshold index. A comprehensive and reasonable indicator system is required to unify multiple enrollment conditions and derive a scientifically objective index standard, providing correct assessment guidance for the enrollment threshold.

This study collects and compiles macro-level policies and admission requirements for migrant children’s schooling in key inflow cities in China. The data are sourced from official provincial and municipal policy documents (2014–2017), the “Law Star” database, the “Bendibao” website, official school announcements, and news reports. Based on policy documents, various admission criteria are extracted, including but not limited to household registration (hukou) requirements, residency proof, and social security contributions, forming the fundamental framework for a Migrant Children’s Compulsory Education Admission Threshold Index in China. Following the methodologies of Zou et al. (32), Guo et al. (102), and De Luca et al. (103), this study employs the Analytic Hierarchy Process (AHP) to systematically quantify the admission threshold. Admission conditions with different units are transformed into 0–1 scores (1 = most lenient, 0 = most stringent), which are then weighted and aggregated to generate provincial-and city-level admission threshold indices. This approach ensures comparability across regions and years.

**Figure 2:**
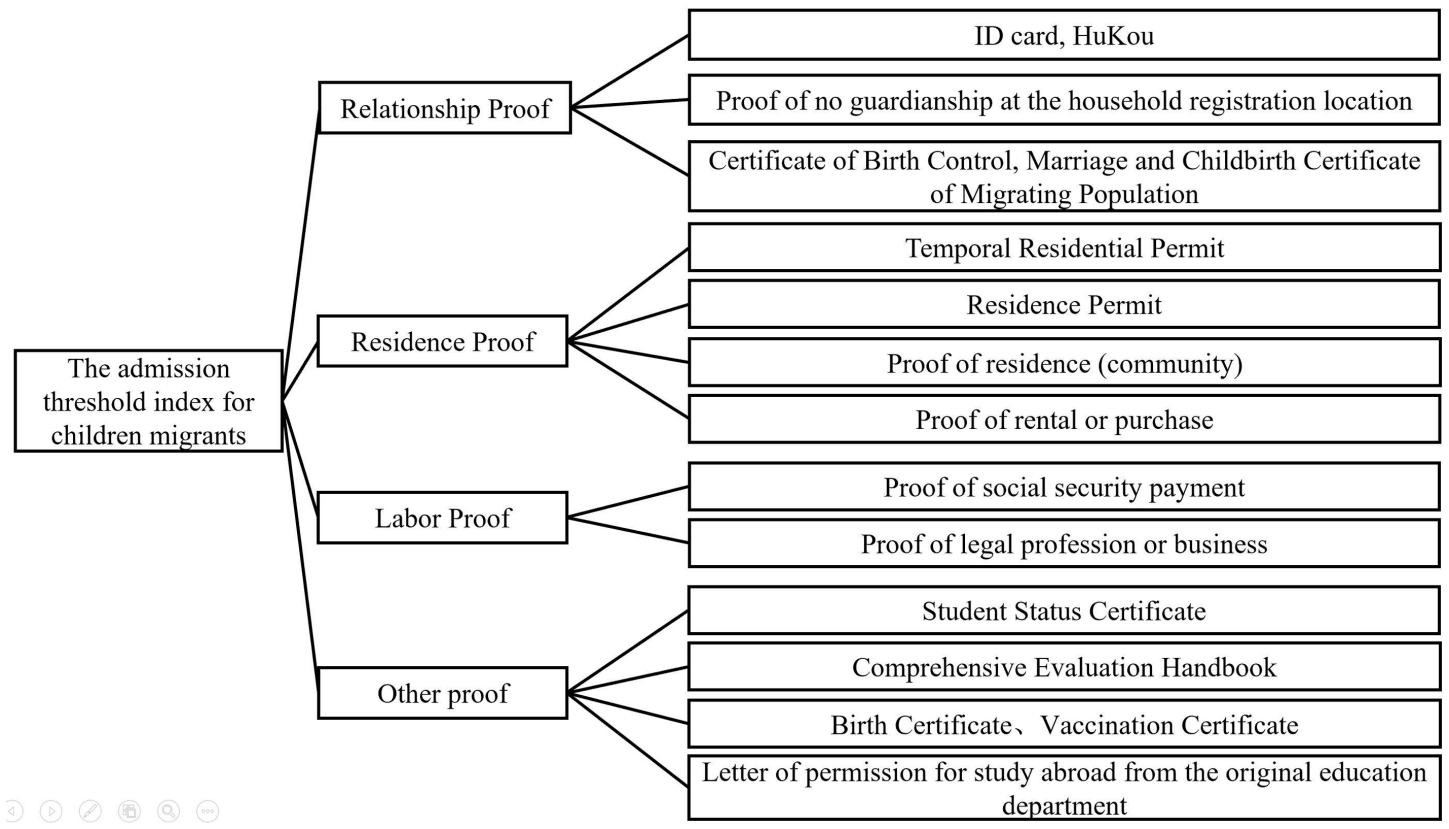
The hierarchical structure of the admission threshold index for migrant children.

The difficulty and status of obtaining various materials for the enrollment threshold of migrant children differ, reflecting the varying importance of different types of documentation for the threshold index. Therefore, assigning weights to various criteria indicators is crucial for the scientific and objective nature of the final results. Based on official policies from various provinces and cities, official school notifications, and news reports on enrollment policies, this study extracted 14 indicator requirements related to enrollment policies. The enrollment condition indicators are categorized into four primary indicators: relationship certificate, residence certificate, employment certificate, and other certificates, with multiple secondary indicators under each primary indicator, aiming to comprehensively reflect the information regarding the enrollment difficulties faced by migrant children, as detailed in Table 1. Based on the above enrollment threshold indicator system, a hierarchical structure model for the enrollment threshold is first established. The hierarchical structure of the enrollment threshold index for migrant children in China is shown in Figure 2. By constructing a judgment matrix and determining the maximum eigenvalue of the judgment matrix, the weight coefficient W is obtained through column normalization. Here, the 1–9 scale method proposed by scholar Thomas L. Saaty is referenced, as shown in Table 4, to quantify the relative importance of the indicators. To avoid subjectivity and uncertainty in the quantification of indicators, consistency was evaluated based on the CR (Consistency Ratio) judgment matrix, and the results passed the consistency test. The final weight results measured by the AHP method are shown in Table 5. The scores for each enrollment condition indicator in various cities are normalized and multiplied by the weights to obtain the enrollment threshold index for the compulsory education stage.

**Table 4:** Saaty’s 1–9 scale method.

Scale	Meaning
1	Two elements are equally important
3	The former is slightly more important than the latter
5	The former is significantly more important than the latter
7	The former is extremely more important than the latter
9	The former is strongly more important than the latter
2, 4, 6, 8	Intermediate values between the adjacent judgments mentioned above
Reciprocal of the above values	Importance comparison after exchanging the order of the two factors mentioned above

**Table 5:** Enrollment threshold weight results.

Goal layer	Criteria layer	Weight	Indicator layer	Weight
Threshold index for enrollment of migrant children	Relationship Proof	0.1132	ID card, HuKou	0.0189
			Proof of no guardianship at the household registration location	0.0566
			Certificate of Birth Control, Marriage and Childbirth Certificate of Migrating Population	0.0377
	Residence Proof	0.4528	Temporal Residential Permit	0.0943
			Residence Permit	0.1132
			Proof of rental or purchase	0.1509
			Proof of residence (community)	0.0943
	Labor Proof	0.2075	Proof of legal profession or business	0.0755
			Proof of social security payment	0.1321
	Other Proof	0.2264	Student Status Certificate	0.0566
			Letter of permission for study abroad from the original education department	0.0566
			Birth Certificate、Vaccination Certificate	0.0566
			Comprehensive Evaluation Handbook	0.0189
			Other conditions	0.0377

#### 4.2.3 Control variable

Regarding the selection of control variables, in order to reduce the interference of other possible factors and focus on the net impact of the enrollment difficulties faced by migrant children on household consumption, we refer to macro consumption theory and existing literature (104–106). The model controls for micro variables such as parental individual characteristics and family circumstances, as well as macro variables related to urban characteristics that may affect household consumption. We referenced existing research on the household consumption of the migrant population and controlled for factors including the personal characteristics of the household head, the personal characteristics of the spouse, and family demographic characteristics. The personal characteristics of the household head include gender, age, and employment status, while the personal characteristics of the spouse include age and household registration status. Family demographic characteristics include the number of family members. Among these, the gender of the household head is coded as male = 1 and female = 0. The ethnicity of the household head is coded as Han = 1 and minority = 2. The household registration status of the spouse is coded as agricultural = 1, non-agricultural = 2, and others = 3.

Since the enrollment policies for migrant children are primarily implemented in cities with population inflows, which are generally provincial capitals or economically developed areas, including city-level variables in the regression can also help reduce bias to some extent. Considering that cities with different administrative statuses and economic conditions may face varying public expenditure burdens, there may be differences in educational fiscal expenditure patterns. We controlled for the proportion of educational expenditure to total fiscal expenditure (gefeper), the regional GDP growth rate (gdpyoy), and per capita regional GDP (ln_gdpct) to quantify various aspects of the economic development level and growth rate of local municipalities, with per capita GDP taken in logarithmic form in the regression. Since service production and consumption occur simultaneously, the proportion of the secondary industry in GDP (Secondus) can capture the service supply bias brought about by the development of the urban service industry, eliminating supply-side influences and revealing demand-side factors. The specific descriptive statistical results can be seen in Table 3.

### 4.3 Model design


(1) $$\begin{array}{c} \mathrm{Lnco}n_{ict}=\beta_{0}+\beta_{1}\mathrm{entrance}\_\mathrm{inde}x_{ict}*\mathrm{child}\_\mathrm{ag}e_{ict}+\\ \;\beta_{2}\mathrm{entranc}{e_{\mathrm{index}}}_{ict}+\beta_{3}\mathrm{chil}{d_{\mathrm{age}}}_{ict}+\beta_{4}{Ζ}_{ict}+\\ \;\beta_{5}\mu_{ict}+\delta_{c}+\theta_{t} \end{array}$$

Due to the presence of unobservable characteristics at both the household and macroeconomic levels, this study employs the Ordinary Least Squares (OLS) method in the baseline regression model to mitigate omitted variable bias. A two-way fixed effects model is used to control for region-specific fixed factors and time trends, improving the accuracy of the estimates. By incorporating interaction terms, the model captures how admission thresholds affect household consumption decisions differently based on children’s ages. This reduces omitted variable bias stemming from age heterogeneity and enhances the identification of the dynamic effects of admission thresholds across different age groups, thereby strengthening causal inference. To ensure the reliability of the hypothesis testing results, multicollinearity tests were conducted, confirming that multicollinearity is not a significant concern. To further examine the heterogeneity of the data, subgroup regressions were performed based on migration range, number of children, and settlement intentions, allowing for an in-depth analysis of differential impacts across various group.

The regression uses the Ordinary Least Squares (OLS) method and employs a two-way fixed effects model to eliminate the influence of regional fixed factors and time trends. Therefore, Equation 1 is constructed, where the subscripts (i), (c), and (t) represent households, cities, and years, respectively. The explanatory variables $\mathrm{entrance}\_{\mathrm{index}}_{ict}\;\mathrm{and}\;\mathrm{child}\_\mathrm{ag}e_{ict}$ represent the enrollment threshold for migrant children and the age of the children, respectively. If a household has multiple children under the age of 12, the age of the oldest child is used. The dependent variable $con_{ict}$ represents household consumption expenditure, ${Ζ}_{ict}$ represents a series of control variables, $\delta_{c}$ is the city fixed effect, $\theta_{t}$ is the year fixed effect, and $\mu_{ict}$ is the random error term.

In the context of enrollment restrictions during the compulsory education stage, the economic development status of various cities and other consumption influencing factors are closely related to the setting of enrollment thresholds. Using enrollment thresholds as the sole estimation variable in the model may overlook other important variables, leading to an incomplete analysis. Compared to other policy systems, a significant characteristic of enrollment restrictions is that their impact on families becomes increasingly pronounced as children approach the enrollment stage. On one hand, when the period of policy impact is still distant, its expected effects are relatively weak (107); at the same time, the policy itself is subject to dynamic adjustments, and some parents hold expectations that the policy may become more lenient when their children face enrollment (108). Therefore, as children’s grades increase, families’ perceptions and experiences of enrollment restrictions deepen. To more accurately capture the actual impact of enrollment restrictions, we should pay special attention to the interaction effect coefficient between enrollment thresholds and children’s grades. If this coefficient is negative, it indicates that in cities where migrant families reside, the higher the enrollment threshold for out-of-town students during the compulsory education stage, the greater the reduction in household consumption as children’s grades increase, demonstrating that enrollment restrictions significantly suppress the consumption effects for migrant families.

To address the endogeneity issue in the baseline regression model, this study employs the two-stage least squares (2SLS) method, using instrumental variables (IV) to mitigate potential endogeneity concerns. This approach effectively addresses endogeneity by satisfying relevance (correlation with the endogenous variable) and exogeneity (exclusion from the error term). Furthermore, the instrumental variable method helps overcome data limitations by addressing omitted variable bias, conducting weak instrument tests, and performing over-identification tests to validate the instrument’s suitability. The chosen instrument, average_ed (the average level of educational fiscal expenditure in surrounding cities within the same province, excluding the local region), is likely correlated with local school admission thresholds but does not directly affect household consumption. This enhances the validity and robustness of the instrumental variable.

## 5 Empirical results

### 5.1 Baseline regression results

The baseline regression first uses the logarithm of household consumption expenditure of the migrant population as the dependent variable, employing OLS to estimate the impact of the enrollment threshold during the compulsory education stage on household consumption expenditure. In Table 6, Columns (2) to (4) in the Table 6 sequentially include household characteristics, urban characteristics, and fixed effects for cities and years based on Column (1) using a stepwise regression method. The $R^{2}$ increases from 0.002 in Column (1) to 0.164 in Column (4), indicating that the model’s explanatory power for changes in household consumption has improved by adding control variables and clustering effects. The results show that the regression coefficient of the interaction term between the enrollment threshold and children’s age is significantly negative, meaning that as children’s age increases, the negative effect of the enrollment threshold on household consumption of the migrant population becomes greater.

**Table 6:** Results of baseline regression.

Variable	Ln (Total consumption)	Ln (Total consumption)	Ln (Total consumption)	Ln (Total consumption)
entrance_index*childage	−0.002***	−0.003***	−0.002***	−0.003***
	(−3.77)	(−5.04)	(−4.10)	(−5.71)
childage	−0.001**	−0.001**	−0.009***	−0.006***
	(−2.01)	(−2.10)	(−13.30)	(−9.39)
entrance_index	0.048***	−0.006	−0.003	0.044
	(11.30)	(−1.60)	(−0.81)	(0.13)
genderflo		0.005*	0.007***	0.009***
		(1.78)	(2.60)	(3.32)
ageflo		−0.001***	0.000	0.000
		(−3.36)	(0.72)	(0.04)
nationflo		−0.090***	−0.094***	−0.072***
		(−18.40)	(−19.25)	(−14.20)
agespo		−0.002***	−0.000	−0.000
		(−4.22)	(−0.68)	(−1.09)
hukouspo		0.225***	0.218***	0.200***
		(95.57)	(93.03)	(85.49)
famsize		0.120***	0.131***	0.131***
		(93.20)	(100.60)	(101.03)
gefeper		−0.147***	−0.364***	0.483***
		(−7.89)	(−8.26)	(6.76)
gdpyoy		0.004***	−0.004***	−0.003***
		(6.10)	(−6.71)	(−2.71)
ln_gdpct		0.283***	0.255***	0.072***
		(78.61)	(68.09)	(3.81)
Secondus		−0.003***	−0.002***	−0.000
		(−28.27)	(−21.42)	(−0.56)
Constant	8.125***	4.588***	4.890***	6.730***
	(1,867.63)	(103.20)	(102.96)	(17.93)
Fixed Effects for Inflow Cities			Control	Control
Fixed Effects for Year				Control
Observations	200,947	200,947	200,947	200,947
R-squared	0.002	0.121	0.130	0.164

***, **, * represent significance levels of 1, 5, and 10%, respectively; the values in parentheses are robust standard errors.

### 5.2 Robustness check

To ensure the accuracy of the previous research results, this paper conducted robustness checks using the following four methods.

#### 5.2.1 Replacing the dependent variable

Replace the dependent variable with consumption expenditure after subtracting housing expenses, per capita household consumption, and total consumption that includes accommodation and meals, respectively, to re-measure household consumption expenditure. The results in Table 7, Columns (1) to (3), indicate that the regression results after replacing the dependent variable are consistent with the conclusions drawn from the baseline regression analysis, further confirming the positive effect of the enrollment threshold policy on promoting household consumption among the migrant population.

**Table 7:** Results of the robustness test (1).

Variable	(1)	(2)	(3)
	Ln (Excluding housing)	Ln (Per capita consumption)	Ln (Room and board included)
entrance_index*childage	−0.002***	−0.003***	−0.003***
	(−2.58)	(−5.45)	(−5.45)
childage	−0.003***	−0.009***	−0.009***
	(−4.11)	(−13.19)	(−13.19)
entrance_index	−0.015	0.142	0.142
	(−0.04)	(0.40)	(0.40)
Constant	6.561***	6.864***	6.864***
	(15.77)	(17.86)	(17.86)
Control variable	YES	YES	YES
FE	YES	YES	YES
Observations	200,775	200,947	200,947
R-squared	0.122	0.163	0.163

***, **, and * indicate significance levels of 1, 5, and 10%, respectively; the values in parentheses represent robust standard errors.

#### 5.2.2 Treatment

To avoid the influence of extreme values in income and consumption data on the estimation results, we applied trimming treatment to household total consumption expenditure and household income at the lower and upper 1 and 2%. Data below the 1st percentile and above the 99th percentile were replaced with the 1st and 99th percentiles, respectively. The regression results in Table 8, Columns (1) to (4), remain consistent with the baseline regression results, indicating that the exclusion of outliers in consumption and income does not affect the fundamental conclusion that a relaxed enrollment threshold has a promoting effect on household consumption. The empirical results are relatively robust.

**Table 8:** Results of the robustness test (2).

	(1)	(2)	(3)	(4)	(5)	(6)
ln (Total consumption)	Consumption winsorization 1%	Consumption winsorization 2%	Income winsorization 1%	Income winsorization 2%	Control Hukou threshold	Excluding sample of municipality
entrance_index*childage	−0.003***	−0.003***	−0.003***	−0.003***	−0.003***	−0.002***
	(−5.95)	(−5.59)	(−5.71)	(−5.71)	(−5.55)	(−3.79)
childage	−0.006***	−0.005***	−0.006***	−0.006***	−0.006***	−0.005***
	(−9.50)	(−9.10)	(−9.39)	(−9.39)	(−9.50)	(−7.30)
entrance_index	0.014	−0.008	0.044	0.044	0.043	0.168
	(0.04)	(−0.03)	(0.13)	(0.13)	(0.12)	(0.41)
Hukou_threshold					0.076***	
					(3.81)	
Constant	7.096***	7.277***	6.730***	6.730***	6.576***	7.191***
	(20.74)	(21.86)	(17.93)	(17.93)	(17.42)	(17.66)
Control variable	YES	YES	YES	YES	YES	YES
FE	YES	YES	YES	YES	YES	YES
Observations	197,259	195,603	200,947	200,947	200,947	163,783
R-squared	0.153	0.145	0.164	0.164	0.164	0.119

***, **, and * indicate significance levels of 1, 5, and 10%, respectively; the values in parentheses represent robust standard errors.

#### 5.2.3 Consider other policies during the same period

During the implementation of the education policy for migrant children, other concurrent policies may also impact the consumption of the migrant population. For example, after obtaining a local household registration, families may benefit from the elimination of labor market discrimination and gain higher welfare protections, effectively increasing household consumption. Therefore, this paper includes the household registration threshold under China’s household registration system to control for the impact of varying difficulties in obtaining local registration on the consumption of migrant families. The regression results in Table 8, Column (5) show that after controlling for relevant policies affecting the migrant population, the education policy for migrant children still significantly influences the consumption of the migrant population, further demonstrating the robustness of the conclusions in this paper.

#### 5.2.4 Exclude the influence of municipality

This paper excludes household samples from the four municipalities: Beijing, Shanghai, Tianjin, and Chongqing. China’s municipalities directly under the central government are directly governed by the central government and enjoy more abundant educational resources. At the same time, given the significant differences in economic development levels, resident income levels, the completeness of social security systems, and population structure characteristics between these municipalities and other non-municipal cities, these factors may act as potential confounding variables for public education and family consumption, leading to systematic bias in the results of the regression analysis. Therefore, this paper excludes household samples from the municipalities to examine the impact of lowering the enrollment threshold on household consumption. The results in Table 8, Column (6) indicate that the estimated results in this paper are robust and reliable.

#### 5.2.5 Exclude other city-level influencing factors

Excluding the influence of other city-level factors, considering that macroeconomic factors at the city level, such as industrial structure and financial development, may be related to admission thresholds in different cities and could have a differential impact on the consumption level of migrant households as children’s ages change. To eliminate the impact of such factors, an interaction term between the child’s grade level and city-level macro variables was included. The specific results are shown in Table 9, where it is found that the coefficient of the interaction term between admission threshold and child’s age remains significantly negative, and the baseline results are robust.

**Table 9:** Results of the robustness test (3).

ln (Total consumption)	(1)	(2)
	Add an interaction item between the child’s grade and the city-level variables	
	The proportion of employees in the tertiary industry	Loan balance of financial institutions / GDP
entrance_index*childage	−0.002***	−0.002***
	(−2.95)	(−2.95)
entrance_index* city-level variable	−0.000	−0.000
	(−1.25)	(−1.25)
childage	−0.002**	−0.002**
	(−2.57)	(−2.57)
entrance_index	−0.017	−0.017
	(−0.04)	(−0.04)
Constant	6.576***	6.576***
	(16.14)	(16.14)
Control variable	YES	YES
FE	YES	YES
Observations	196,413	196,413
R-squared	0.125	0.125

***, **, and * indicate significance levels of 1, 5, and 10%, respectively; the values in parentheses represent robust standard errors.

### 5.3 Endogeneity test

This paper studies the impact of the enrollment threshold for compulsory education on the consumption of the migrant population. Since an individual cannot influence the overall enrollment policy of the host city, there is no logical reverse causality in this context. However, there is a concern regarding omitted variable bias due to the heterogeneity of cities with migrant populations, which may lead to unobserved variables being overlooked. This paper employs an instrumental variable approach combined with the two-stage least squares (2SLS) method to address potential endogeneity issues arising from omitted variables, ensuring the accuracy of parameter estimates and the reliability of research conclusions.

The peer effect is regarded as one of the endogenous factors influencing residents’ consumption behavior in accordance with the theory of relative consumption. It reflects the consumption adjustment response individuals make when confronted with changes in the proximate social environment. Maurer and Meier (71) have highlighted the existence of a notable synchrony between individual consumption behavior and the consumption patterns of the group to which they belong. This article refers to the research by Li and Fang (108) and selects “Average level of financial expenditures on education in neighboring cities in the same province” (average_ed) as the instrumental variable. Other cities in the same province usually have similar preferences for public education supply, so the education fiscal levels of these cities can effectively reflect the education supply levels of the region, meeting the relevance requirement for the instrumental variable. Although the public education policies of various cities in the same province may have spatial proximity effects, the education policies of other cities are unlikely to directly influence the consumption levels of local residents. In summary, the selection of this instrumental variable in this article is theoretically feasible.

The following is the estimation of the model using the 2SLS method, with the main results reported in Table 10. As shown in Table 11, the estimated coefficients of the instrumental variable are significant; the estimated coefficients in the second stage of Table 10 are all significantly positive. This indicates that after addressing the endogeneity bias in the use of the instrumental variable, a decrease in enrollment thresholds will release the consumption of migrant families. Additionally, the Wald test results are significant, and the first-stage *F*-value is greater than 10, demonstrating that the estimation results are relatively robust. Thus, it can be seen that the instrumental variable selected in this article has passed the validity test, and the model estimation results are robust. After controlling for endogeneity issues, the regression coefficient of the core explanatory variable significantly increases and is significant at the 1% level, further confirming the promoting effect of lowering enrollment thresholds on the consumption of migrant families.

**Table 10:** Results of the endogeneity test (1).

Variable	Independent variable (ln_total consumption)
entrance_index*childage	−0.021***
	(0.005)
entrance_index	0.135***
	(0.038)
childage	0.014***
	(0.004)
Constant	4.589***
	(0.045)
Observations	20,0947

***, **, * represent significance levels of 1, 5, and 10%, respectively; robust standard errors are reported in parentheses.

**Table 11:** Results of the endogeneity test (2).

Variable	First-stage regression results
average_ed	1.434***(0.03)
First-stage F statistic	2227.93
Second-stage F statistic	2133.84
Under-identification test (Anderson canon. Corr. LM statistic)	2203.65 *p*-value = 0.0000
Weak instrument test (Cragg-Donald Wald F statistic)	2227.93

Standard errors are in parentheses; *, **, *** indicate significance at the 10, 5, and 1% levels, respectively; the Anderson canonical correlation LM statistic is the statistic for the under-identification test; the Cragg-Donald Wald F statistic is the test statistic for weak instruments; the Sargan statistic is 0.000.

### 5.4 Heterogeneity analysis

#### 5.4.1 Range of mobility

The range of mobility reflects the residential preferences of the migrant population and influences their willingness to integrate (109). This paper categorizes the mobility distance of household heads into three types: inter-provincial mobility, intra-provincial inter-city mobility, and intra-city mobility, in order to analyze the impact of education policies for migrant children on the consumption of families with different mobility distances. The specific regression results are shown in Table 12 columns (1) to (3). The changes in enrollment thresholds have a significant positive effect on the consumption of families with “inter-provincial mobility” and “intra-provincial mobility,” with the effect on “intra-provincial mobility” families being significant at the 1% level and on “inter-provincial mobility” families at the 5% level. This indicates that changes in the enrollment threshold for migrant children can significantly affect the consumption of the migrant population across different mobility distances.

**Table 12:** Results of heterogeneity analysis (1).

Variables	Mobility distance			Number of children	
	Inter-provincial mobility	Intra-provincial mobility	Intra-city mobility	Only-child	Non-only-child
entrance_index*childage	−0.002**	−0.003***	−0.003*	−0.005***	−0.001
	(−2.35)	(−3.38)	(−1.65)	(−4.90)	(−1.45)
childage	−0.007***	−0.005***	−0.002	−0.015***	−0.000
	(−6.81)	(−5.55)	(−1.46)	(−14.61)	(−0.31)
entrance_index	−0.149	0.397	−1.109	−0.217	0.452
	(−0.28)	(0.84)	(−0.97)	(−0.49)	(0.82)
Constant	6.052***	5.060***	8.694***	6.391***	6.963***
	(9.67)	(7.56)	(10.09)	(11.78)	(12.46)
Control variable	YES	YES	YES	YES	YES
FE	YES	YES	YES	YES	YES
Observations	110,072	69,665	21,137	90,902	110,045
R-squared	0.194	0.147	0.161	0.190	0.150

***, **, and * represent significance levels of 1, 5, and 10%, respectively; values in parentheses are robust standard errors.

Under the context of population mobility, as school admission policies for migrant children continue to adjust, intra-provincial migrant populations, being within the same provincial administrative unit, can more easily align with the household registration system (hukou) and overcome institutional barriers. However, inter-provincial migration still faces fragmented policies, which hinder the reduction of precautionary saving motives. Moreover, in regions with similar cultural and lifestyle backgrounds, migrants are more likely to establish strong social and emotional networks, integrating more easily into local acquaintance circles and kinship networks, thereby enhancing their sense of identity and family security. Studies have shown that groups with a stronger sense of identity tend to exhibit higher consumption levels (110). Therefore, the provision of basic education has a more significant impact on increasing consumption among intra-provincial migrant families. This phenomenon highlights the effectiveness of regional policy coordination. It suggests that inter-provincial recognition of public education resources and identity status should be strengthened to reduce administrative burdens and procedural complexities in cross-province school transfers. Additionally, it reveals the crucial role of social support networks in economic behavior (64). Policies should focus on enhancing educational support within social networks, such as establishing community education service centers, to lower the information acquisition costs for migrant families.

#### 5.4.2 Whether the family is an only-child family

Although the global pension insurance system is becoming increasingly sophisticated, the issue of older adults care remains a key factor concerning family welfare and stability. This paper divides families of the migrant population into two groups based on the number of children: only-child families and non-only-child families, with the results shown in Table 12. It can be observed that, for only-child families, the absence of siblings to share the future costs of older adults care and living expenses leads them to prefer precautionary savings and place greater emphasis on their children’s education (111). They hope that through the accumulation of human capital, the family’s future economic situation will improve. If their children are able to achieve higher incomes, the family can secure more resources for older adults care.

At the same time, parents’ expectations for education are also a key factor influencing household consumption. Under the trend of low birth rates, children’s educational opportunities are regarded as a crucial pathway for families to participate in social competition. The “all-or-nothing” nature and irreversibility of education investment drive only-child families to devote substantial resources to creating an optimal growth environment for their children, further reinforcing their precautionary saving motives. As a result, within only-child families, reductions in public education costs and relaxation of education policies at the compulsory education stage can significantly enhance household consumption, leading to the spillover effects of education policy dividends and highlighting the profound impact of education policies on household economic behavior. To address this, a differentiated education subsidy system for only-child families could be implemented. For example, policymakers could introduce a targeted education resource subsidy system, where education subsidies or insurance premiums are directly linked to policy benefits, thereby reducing cost fluctuations during children’s educational stages.

#### 5.4.3 Intention to stay

The willingness to stay reflects the expectations of the migrant population regarding living and working in the destination area, and it indicates their sense of identity and degree of integration into that region. As the institutional barriers to public education services are gradually eliminated with the relaxation of education policies for migrant children, this significantly influences the future education expectations of families with a strong willingness to stay. The regression results in Table 13 report the impact of residency intention on the consumption of the migrant population. It shows that families with a willingness to stay are more inclined to achieve their educational goals through consumption, thereby unlocking the local consumption potential (112).

**Table 13:** Results of heterogeneity analysis (2).

Variable	Willingness to stay		Whether children migrate	
	Willingness to stay	No willingness to stay	Children migrate	Children left behind
entrance_index* childage	−0.003***	−0.002*	−0.001**	−0.004***
	(−4.95)	(−1.80)	(−2.03)	(−3.37)
childage	−0.008***	−0.004***	−0.003***	−0.009***
	(−10.03)	(−3.07)	(−3.90)	(−6.36)
entrance_index	0.237	−0.764	−0.223	0.991
	(0.62)	(−0.97)	(−0.60)	(1.26)
Constant	6.364***	7.935***	6.662***	5.061***
	(14.71)	(10.46)	(15.79)	(6.10)
Control variable	YES	YES	YES	YES
FE	YES	YES	YES	YES
Observations	145,061	55,886	140,380	43,776
R-squared	0.154	0.142	0.172	0.136

***, **, and * represent significance levels of 1, 5, and 10%, respectively; values in parentheses are robust standard errors.

This reflects that when children benefit from more inclusive public education policies, migrant families with residency intentions tend to develop a stronger sense of urban belonging. This leads to a deeper integration into social networks and enhances identity-driven consumption behaviors. At the same time, improving education policies is also perceived as a signal of government commitment, providing families with residency intentions greater certainty about the future. As a result, they reduce precautionary savings and increase consumption confidence. Globally, the trend of family migration is becoming more apparent, and household heads with strong residency intentions are more willing to relocate their entire families to the destination area. This not only enhances their integration and sense of happiness in the new environment but also boosts their household consumption levels. Therefore, the interaction between the migrant population’s residency intention and education policies, in the context of globalization, has become a critical factor in unleashing domestic demand and promoting local economic development.

#### 5.4.4 The situation of migrant children

Living apart from their children for extended periods not only increases the concerns of migrant working parents and lowers their subjective well-being, but it also negatively affects the physical and mental health of the children left behind. The regression results in Table 13 demonstrate the impact of whether children migrate with the family on household consumption. The lowering of school admission thresholds for migrant children provides a crucial educational foundation for migrant families in their destination cities. When migrants secure stable jobs and housing in urban areas, they are more likely to enroll their children locally rather than sending them back to their hometowns for education.

This decision is driven by reduced future education costs and greater stability in their children’s education, allowing them to benefit from a higher-quality educational environment. However, for families left behind in their hometowns, the spillover benefits of education policies are weaker, resulting in a more limited impact on household consumption. Family migration plays a critical role in the social integration of the migrant population, which in turn reduces the pressure to engage in precautionary savings and releases household consumption potential. It also helps alleviate the relative deprivation children may feel in accessing public education services. As a result, families tend to increase their spending on education and other areas for children not living with them to improve their quality of life and enhance their human capital (113). Therefore, education policies for migrant children should aim to reduce institutional friction costs by enhancing cross-regional coordination, thereby minimizing implementation inefficiencies associated with education policies.

#### 5.4.5 The situation of income

This paper further analyzes the heterogeneity in household characteristics by dividing the full sample of household income into tertiles based on annual income, in order to examine the differential impact of school enrollment thresholds on the consumption levels of migrant population families across different income groups. According to the regression results in Table 14, it is found that the effect of the enrollment threshold on the consumption levels of families in the lowest 33% and middle 33% income brackets is significantly negative at the 1% significance level, while the effect on high-income families is not significant.

**Table 14:** Results of heterogeneity analysis (3).

Variable	Household income situation			Nature of the household head’s work	
	High income	Middle income	Low income	Non-public sector	Public sector
entrance_index*childage	−0.001	−0.002***	−0.007***	−0.003***	−0.005**
	(−0.92)	(−2.98)	(−6.26)	(−5.05)	(−2.03)
childage	−0.003***	−0.003***	−0.003***	−0.006***	−0.016***
	(−3.17)	(−3.77)	(−2.60)	(−7.57)	(−6.01)
entrance_index	0.271	0.879	0.288	−0.012	0.107
	(0.55)	(1.47)	(0.60)	(−0.03)	(0.12)
Constant	7.716***	7.481***	7.174***	6.781***	5.913***
	(16.29)	(11.98)	(9.59)	(15.05)	(4.17)
Control variable	YES	YES	YES	YES	YES
FE	YES	YES	YES	YES	YES
Observations	64,467	64,755	64,850	157,146	11,931
R-squared	0.096	0.118	0.182	0.163	0.251

***, **, and * represent significance levels of 1, 5, and 10%, respectively; values in parentheses are robust standard errors.

These regression results reflect the income redistribution effect of public education support, which alleviates the financial burden on families and reduces income disparities. This is particularly effective in reducing the spending pressure on low-and middle-income families, thereby enhancing the welfare of migrant population households and, to some extent, promoting social mobility and class advancement. On the other hand, it also reflects the impact on social mobility. For low-and middle-income families, a lower school enrollment threshold can significantly enhance their prospects for upward mobility and human capital development, thereby driving an increase in household consumption levels. As the global economic level generally improves, the marginal effect of public education supply shows a diminishing trend. This phenomenon indicates that high-income groups have nearly reached saturation in meeting their basic living needs. Consequently, the proportion of social education support in these families’ total income has relatively decreased, limiting its marginal effect in further improving the quality of life. This also highlights the importance of the effectiveness and targeting of public education policies in promoting social equity and driving economic development. Therefore, a targeted education subsidy system can effectively ensure that low-and middle-income families benefit from policy dividends. This approach guarantees that educational funding reaches the intended migrant children’s families directly, preventing excessive resource allocation that could lead to inequities.

#### 5.4.6 Nature of work

Based on ownership, workplaces in China can generally be divided into public sector units and non-public sector units. Jobs in public sector units often provide more stable income sources and welfare benefits. This paper categorizes the work units of household heads based on the nature of their employers into public sector and non-public sector units, meaning whether the household head works for government agencies, public institutions, or state-owned and state-controlled enterprises. The regression results are shown in Table 14. columns (4) and (5), which report the impact of migrant children’s education policies on household consumption levels for heads of households working in public sector units and non-public sector units, respectively. The adjustment of school enrollment thresholds has a statistically significant impact at the 1% level for households with heads working in non-public sector units, and at the 5% level for those with heads working in public sector units.

This phenomenon may be due to the following reasons: 1. Families within the public sector are more likely to meet the more challenging requirements of school enrollment thresholds, such as local household registration and stable employment, allowing them to benefit from higher levels of social security, including medical insurance, pension insurance, and housing provident funds. This provides them with a stronger sense of security when facing future economic fluctuations and uncertainties. 2. In contrast, non-public sector families (such as individual businesses, private enterprises, and joint-stock or partnership enterprises) generally have lower levels of social security and stability due to the nature of their employers. Thus, the compulsory education policies for migrant children can serve as a “safety net” and facilitate income redistribution, providing basic educational guarantees for the children of the migrant population and reducing the precautionary savings needs of low-mobility families, thereby releasing more consumption potential. Additionally, the long-term income of public sector families tends to be more stable, while non-public sector families often face weaker material conditions. The improvement in basic welfare levels provides these families with stronger life security, amplifying the effect of changes in migrant children’s education policies on boosting household consumption (Table 14).

## 6 Conclusion and policy implications

Due to the uneven distribution of educational resources across regions, the challenges related to school enrollment policies for compulsory education have had a significant impact on the household consumption of the migrant population. This paper, based on microdata from the China Migrants Dynamic Survey (CMDS) covering 200,947 migrant households from 2014 to 2017, and using a hierarchical model of the school enrollment threshold index, investigates the impact of migrant children’s enrollment policies on household consumption. The overall findings indicate that the reduction of school enrollment thresholds during the compulsory education phase significantly promotes the overall consumption of migrant households. Families experiencing intra-provincial migration, those with only children, families with a willingness to stay, families where children have not migrated with them, middle-and low-income households, and families where the household head works in non-public sector units are more significantly affected.

Based on the above conclusions, this paper puts forward the following three policy recommendations. First, the education issue of migrant children is a microcosm of the large-scale population migration process, revealing the important role of government education policies in strengthening the well-being and sense of fulfillment of the existing migrant population. Therefore, it is necessary to rely on digitalization and intelligence, comprehensively consider the ability to manage educational information and resources, establish a full-chain education tracking system for migrant children, and ensure the controllability of student mobility between “inflow areas and outflow areas.” This will improve the modernization level of the education governance system, help migrant children obtain sufficient support from social education resources, and enhance future human resource development.

Second, barriers to cross-regional mobility for migrant children should be addressed and eliminated through cooperation between multiple social sectors. This would promote lower migration costs for migrant populations and their families, enabling smoother cross-regional mobility. Thus, interregional information sharing and cooperation are encouraged to promote the equalization of public resources such as education in both outflow and inflow areas. This can provide an effective reference for countries with similar residency management methods as China’s household registration system, further safeguarding the rights of migrant populations and migrant children, and promoting their social integration and development in inflow areas.

Third, due to differences in economic, cultural, and social backgrounds within the migrant population, differentiated and continuous policies for migrant population protection need to be developed by both the government and society. This would reduce further imbalances in educational opportunities and resources caused by these differences. Paying attention to educational equity within the group is of great significance for promoting the social integration and long-term development of the entire migrant population. Efforts should be made to ensure that all migrant children have access to equal educational opportunities and quality, addressing the issue of intergenerational poverty transmission on a larger scale and at a deeper level, and ultimately achieving social harmony and stability.

Fourth, region-specific education equity policies should be promoted based on local characteristics. For megacities and economically developed cities, the government should increase the supply of public school seats and relax the residency duration requirements for school enrollment. For medium-sized or emerging cities, a “zero-threshold” enrollment policy should be promoted. This not only stimulates household consumption but also attracts more migrant populations, expanding the local labor market. To address potential fiscal pressures and unequal distribution of educational resources, the central government should implement a dynamic fiscal-sharing mechanism. This would ensure that education funding follows the migrant population, reducing the financial burden on host cities. Additionally, special allowances for teachers in migrant schools should be strengthened to encourage high-quality teaching resources to be directed toward schools with a high concentration of migrant children.

## 7 Limitations

Firstly, this study is based on microdata samples from a questionnaire survey analyzing regional disparities and inequality. Therefore, there may be some subjective errors. Future research should focus more on objective data to eliminate measurement biases. Secondly, due to the limitation of data years, it is difficult to analyze changes in the enrollment policies for migrant children in the most recent stages. If more recent data from the past few years were available, further research could be conducted on the consumption issues of migrant families. In future research, in addition to family economic behavior and public policy support, the study could also explore changes in economic behavior within families under the background of public education policies from cultural and psychological perspectives.

## Data Availability

The original contributions presented in the study are included in the article/supplementary material, further inquiries can be directed to the corresponding author.
